# Sortilin as a Novel Diagnostic and Therapeutic Biomarker in Chronic Lymphocytic Leukemia

**Published:** 2019

**Authors:** Lia Farahi, Fatemeh Ghaemimanesh, Saeideh Milani, Seyed Mohsen Razavi, Mohammad Mehdi Akhondi, Hodjattallah Rabbani

**Affiliations:** 1. Monoclonal Antibody Research Center, Avicenna Research Institute, ACECR, Tehran, Iran; 2. Clinic of Hematology and Oncology, Firoozgar Hospital, Faculty of Medicine, Iran University of Medical Sciences, Tehran, Iran

**Keywords:** Apoptosis, Biomarker, Chronic Lymphocytic Leukemia, Monoclonal antibody, Sortilin

## Abstract

**Background:**

The overexpression of sortilin/neurotensin receptor 3 has previously been reported in various human solid tumors but not in hematological malignancies. Here, we report the overexpression of sortilin in leukemic cells from patients with Chronic Lymphocytic Leukemia (CLL).

**Methods:**

Flow cytometry was used to compare the expression of sortilin in CLL patients (n=52) and healthy individuals (n=26). Also, in vitro apoptosis induction was assessed in CLL Peripheral Blood Mononuclear Cell (PBMCs) following directly targeting of sortilin.

**Results:**

The results showed a significant expression of sortilin on the surface of CLL PBMCs (range from 2.2 to 71.5%) in comparison to healthy individuals (range from 0.03 to 7.4%) (p≤0.0001). The optimal cut-off value of sortilin expression was determined at 7.2% with high sensitivity and specificity. Treatment of leukemic cells with anti-sortilin antibody could induce apoptosis without any effect on normal cells.

**Conclusion:**

Apoptosis induction in CLL cells together with a significant correlation between the expression of sortilin and CD23 represent a possible functional role of sortilin in leukemogenesis of CLL cells. Therefore, sortilin might be considered as a promising novel biomarker in diagnosis, monitoring, and therapy of patients with CLL.

## Introduction

B cell Chronic Lymphocytic Leukemia (B-CLL) is a clonal lymphoproliferative disorder characterized by accumulation of mature-appearing neoplastic lymphocytes with co-expressing CD5, CD19, and CD23 1. The majority of CLL patients live for a long time without any treatment. However, CLL has still to be considered as an incurable disease. Due to low-efficient therapy and risk of side effects, it is often advised waiting until the disease become progressive and the bothersome symptoms appear. Therefore, the discovery of molecular biomarkers enabling us to predict disease progression to start therapeutic interventions would be advantageous 2. Indeed, improvement in our knowledge about the biology of CLL will improve the therapeutic options.

Sortilin is a 95 *kDa* transmembrane glycoprotein with a deregulated expression in many human cancers and also neurological disorders 3–5. Human sortilin is encoded by *SORT1* gene located on chromosome 1 and classified as a member of mammalian vacuolar protein sorting 10p domain (Vps10pD) family 6. As a multifunctional receptor, sortilin mediates transport of proteins such as neurotensin and neurotrophin to cell membrane or lysosomes, directing cell survival and tumorigenesis 7,8.

Several studies have reported that sortilin is deregulated in various human carcinomas including breast 5, colon 3, prostate 9, lung 10 and melanoma 11. We have also previously reported the overexpression of sortilin in ovarian carcinoma using a developed specific mAb (clone 2D8) 12,13. Here, we used this antibody to study the overexpression of sortilin in CLL patients in comparison with healthy individuals. The induction of apoptosis in CLL Peripheral Blood Mononuclear Cells (PBMCs) following 2D8 mAb treatment showed that sortilin may function as a survival factor in CLL. In this study, we attempted to evaluate sortilin/neurotensin receptor as a new biomarker in CLL and also determine the application of anti-sortilin antibody in targeting CLL leukemic cells.

## Materials and Methods

### Patients and healthy individuals

Blood samples were collected from untreated CLL patients (n=52) referred to Firoozgar Hospital (Tehran, Iran) and healthy individuals (n=26). Patients and healthy individuals were informed for the content of study and consented to provide blood sample for research purposes. PBMCs were *isolated* using Ficoll-Paque plus (GE Healthcare, Little Chalfont, UK) according to the manufacturer instructions 14. The study was conducted in accordance with the 1964 Helsinki Declaration and was approved in the ethical committee of Avicenna Research Institute (ARI).

### Cell lines

Cell lines including 232-B4, I83-E95, WA-C3CD5^+^, Jurkat, RPMI 8226, Caov-4 and SKOV3 (National Cell Bank of Iran, Tehran, Iran) as well as Lymphoblastoid Cell Line (LCL) 15 were cultured in RPMI 1640 media (10% FBS) (Gibco, Grand Island, NY) at 37°*C* in a humidified incubator with 5% CO_2_.

### Flow Cytometry

CLL and healthy PBMCs were incubated with 10 *μg/ml* of anti-sortilin antibody clone 2D8 12 oriso type control mAbs (ARI, Tehran, Iran). Afterwards, FITC-conjugated sheep anti-mouse Ig (1:50) (ARI) was added. Primary antibodies were CD5-FITC/CD19-PE, CD23-PE (5 *μl*) (DAKO, Troy, MI), CD38-PE (10 *μl*) and ZAP-70-PE (10 *μl*) (BD Biosciences, San Jose, CA). To analyze the expression of ZAP-70, PBMCs were fixed and permeabilized using Bioscience kit (San Diego, CA). Fluorescence intensity was analyzed using BD Accuri™ C6 Plus flow cytometer (BD Biosciences).

### Immunofluorescent staining

Thirty thousand cells were seeded and fixed with acetone following blocking with 5% normal sheep serum for 30 *min*. The slides were incubated with2D8 or isotype control mAbs (5 *μg/ml*) for 45 *min*. The slides were further incubated with FITC-conjugated sheep anti-mouse Ig (1:50) (ARI). The nuclei were stained with 1 *μg/ml* of 4′,6-Diamidino-2-phenylindole dihydrochloride (DAPI, Sigma). The slides were observed under an Olympus BX51 fluorescent microscope (Tokyo, Japan).

### Apoptosis assay

PBMCs were purified from CLL patients (n=6) and healthy individuals (n=6) under sterile conditions. Cells (4×10^5^) were cultured in 400 *μl* of RPMI-1664 (5% FBS) (Gibco) in 24-well plates for 2 *hr*, and then treated with 10 *μg/ml* of 2D8 or isotype control mAbs. Staurosporine (2 *μM*) (Sigma) was served as positive control. The controls including isotype control mAb, and staurosporine were used simultaneously in other study 16. After 14 to 18 *hr* incubation, cells were removed from the culture medium and stained with Annexin V FITC and Propidium Iodide (PI) (BD Biosciences) followed by FACS analysis using BD Accuri™ C6 Plus flow cytometer. To calculate the percentage of apoptotic cells, the cell percentage in early (*i.e.* Annexin V^+^, PI^−^) and late apoptosis (*i.e.* Annexin V^+^, PI^+^) were added together.

### Statistical analysis

Statistical analysis was performed using Student’s t-test for parametric data and Mann–Whitney U test for nonparametric data. Receiver operating characteristic (ROC) curve was used to determine the optimal cut-off value of sortilin expression. Analyses were performed using GraphPad Prism 6 software and statistical significance was defined as p≤0.05.

## Results

### Sortilin expression in CLL and healthy PBMCs

The expression of sortilin on the cell surface of purified PBMCs from 52 CLL patients (median age 59 years, range 40–81) was compared to 26 healthy individuals (median age 46.5 years, range 25–70). The biological characteristics and immunophenotyping of CLL patients are summarized in table 1.

**Table 1.** Clinical characteristics of CLL patients

**Gender**	N=52
Male/Female	30/22
**Age**	
Median	62
Range	42–85
**Treatment history**	(N)
Prior treatment	0
**Rai stage**	(N)
0 (n=38), I (n=7), II (n=5), III and IV (n=0)	50
**Lymphocyte count**	(10^9^/l )
Median (range)	30 (11–150)
**CD23**	(%)
Mean±SEM	58±3
Median (range)	63 (2–98)
**CD5^+^/19^+^**	(%)
Mean±SEM	77±1.8
Median (range)	78 (47–97)
**ZAP-70**	(N)
Greater than 20%	1
Lower than 20%	51
**CD38**	(N)
Greater than 30%	16
Lower than 30%	36

**Figure 1.**
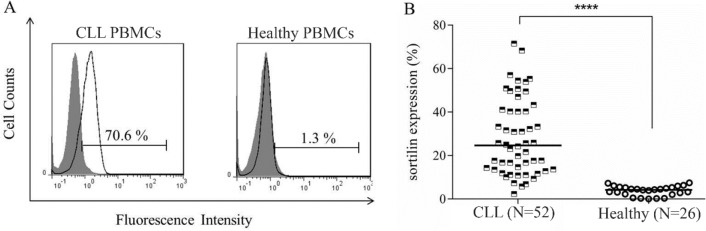
Expression of sortilin in CLL patients and healthy individuals. A) MAb 2D8 specifically detected sortilin on the surface of CLL cells but not healthy PBMCs. The filled gray histogram represents the isotype control mAb staining. B) The scatter plot displays expression level of sortilin in CLL patients in comparison with healthy individuals (****, p≤0.0001). Horizontal line shows the median value of expression.

Flow cytometry results showed that 2D8 mAb specifically detected sortilin on the surface of CLL cells, but not healthy PBMCs (Figure 1A). Results demonstrated that the median expression of sortilin in CLL patients was 24.6% (range from 2.2% to 71.5%) in comparison with healthy individuals which was 4.2% (range from 0.03% to 7.4%) (p≤0.0001) (Figure 1B). However, no significant difference in sortilin expression was observed between progressive and non-progressive cases or even between different stages (0, I and II) of the malignancy. The commercial polyclonal antibody did not detect any cell surface expression of sortilin neither in healthy nor in leukemic cells.

### Determining cut-off value

**Figure 2.**
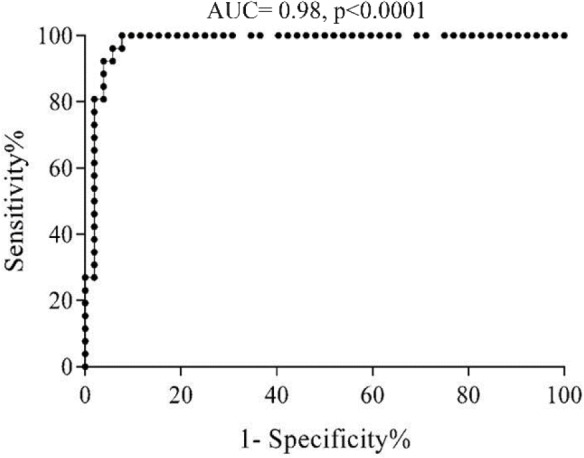
ROC curve shows the trade-off between sensitivity and specificity for cut-off value of sortilin expression in CLL patients. The optimal cut-off value was determined at 7.2% with the sensitivity and specificity of 96.1 and 94.2%, respectively. The Area Under the Curve (AUC) was 0.98.

The cut-off value of sortilin expression in CLL patients was determined at 7.2% (AUC: 0.98, sensitivity: 96.1%, specificity: 94.2%) (p≤0.0001) (Figure 2). Considering cut-off value of sortilin expression (7.2%), 3 out of 52 patients (5.7%) were identified as negative, and only one out of 26 healthy individuals (3.8%) was presented as false positive.

### Immunofluorescent staining

**Figure 3.**
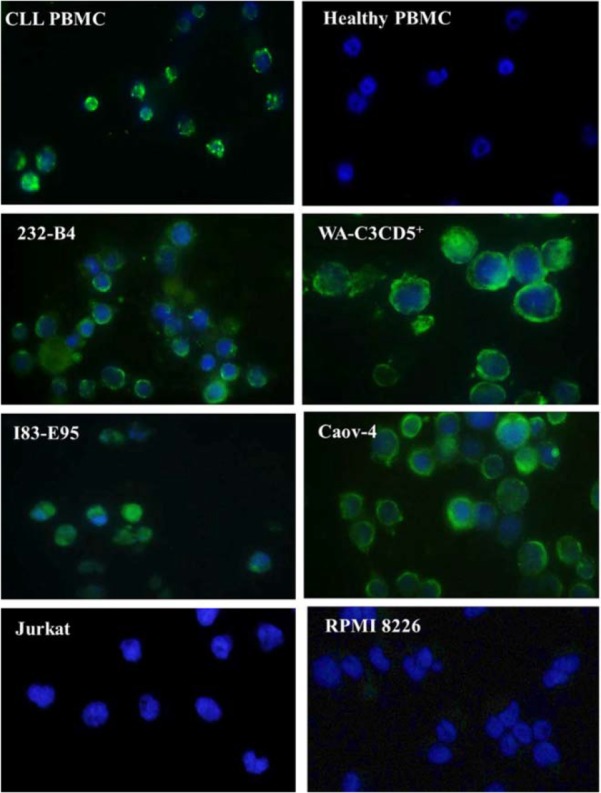
Detection of sortilin expression using immunofluorescent staining. MAb 2D8 detected sortilin (green color) in CLL but not in healthy PBMCs. Sortilin was also detected in CLL cell lines (232-B4, WA-C3CD5^+^ and I83-E95). Caov-4 ovarian cancer cell line was used as positive control. Nuclei were counterstained with DAPI (blue color).

The expression of sortilin in CLL patients was also investigated by immunofluorescent staining. Figure 3 shows that sortilin is expressed in leukemic cells but not in healthy PBMC. MAb 2D8 also detected sortilin in CLL cell lines including 232-B4, WA-C3CD5+ and I83-E95 (Figure 3). Caov-4 ovarian carcinoma cell line was used as positive control 12. However, sortilin expression was not detected in acute T cell leukemia (Jurkat) cell line as negative control.

### Western blot

Cells were lysed in a lysis buffer (1% Triton X-100, 50 *mM* Tris, pH=7.4, 150 *mM* NaCl, 5 *mM* EDTA, 1 *mM* NaF, 20 *mM* Na_4_P_2_O_7_, 1% glycerol, 0.1% sodium dodecyl sulfate) containing 10% phosphatase inhibitor and 1% protease inhibitor cocktail. Following addition of dithiothreitol (5 *mM*) to samples, 15 *μg* of cell lysate were loaded to each well of 10% Sodium dodecyl sulfate polyacrylamide gel electrophoresis (SDS-PAGE) with subsequent electro-transferring to PVDF membrane. The membranes were probed with primary antibodies including 2D8 mAb (10 *μg/ml*) 12, rabbit anti-sortilin pAb (1 *μg/ml*) (Abcam, Cambridge, UK) or anti-β actin mAb (1 *μg/ml*) as internal control 17. HRP-conjugated sheep anti-mouse Ig (1:2500) or anti-rabbit Ig (1:3500) (ARI) were added. The membranes were developed using an enhanced chemiluminescence system (ECL, GE Healthcare) according to the manufacturer’s instructions.

### Correlation between sortilin and CD23

**Figure 4.**
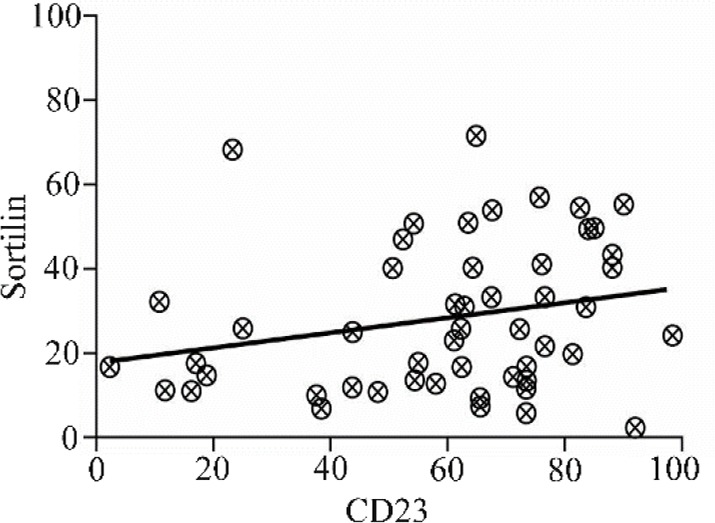
Scatter diagram showing correlation between sortilin and CD23 in PBMCs isolated from CLL patients. The trend line facing upwards shows a positive correlation between the two parameters (r=0.27, p=0.045).

A significant correlation was observed between sortilin and CD23expression in leukemic cells (r=0.27, p=0.045) (Figure 4). However, no correlation between sortilin and other CLL immunophenotypic markers including CD38 and ZAP70 was identified (data not shown).

### Apoptosis assay

**Figure 5.**
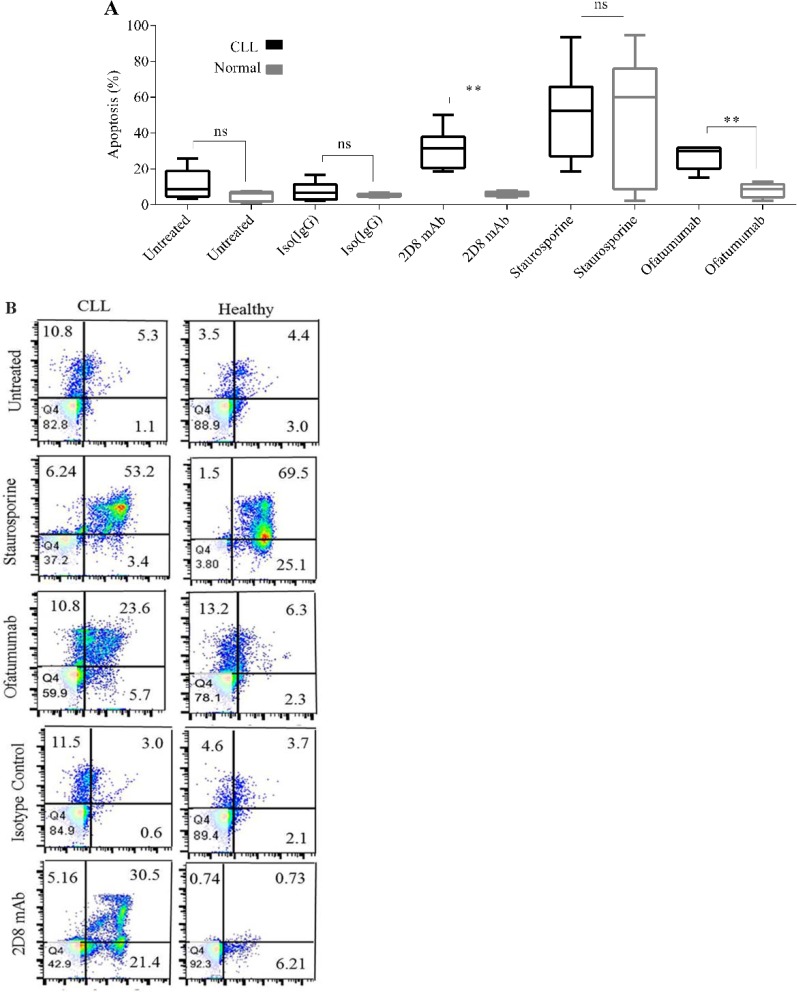
Annexin V apoptosis assay in CLL and healthy PBMCs. A) Box plot represents the frequency (%) of apoptotic cells induced by2D8 mAb in comparison with isotype control mAb (negative control) or staurosporine (positive control) in CLL patients (n=6) (black color) or healthy individuals (n=6) (gray color). Boxes show values between the 25^th^ and 75^th^ percentiles. The horizontal line within the box represents the median value. B) Dot plot diagram represents apoptosis induction in one CLL PBMC in comparison with one healthy PBMC. To calculate the percentage of apoptotic cells, the amount of cell percentage in early (i.e. Annexin V+, PI−) and late (i.e. Annexin V+, PI+) stages of apoptosis was added together. ** means p≤0.01. ns stands for not significant.

Apoptosis induction following incubation with 2D8 mAb was compared between CLL (n=6) and healthy (n=6) PBMCs (Figure 5). Figure 5A is the representative of one out of six individuals in each group of CLL patients and healthy controls. Results demonstrated that 2D8 mAb induced apoptosis in CLL cells (median: 31.5%) with very low effect on healthy individuals (median: 5.6%) (p≤0.01) (Figure 5B). Isotype control mAb did not induce significant apoptosis in CLL (6.5%) or healthy individuals (5.1%). Staurosporine, as positive control, induced apoptosis in both CLL (52.5%) and healthy PBMCs (60%) (p>0.05).

## Discussion

**Figure 6.**
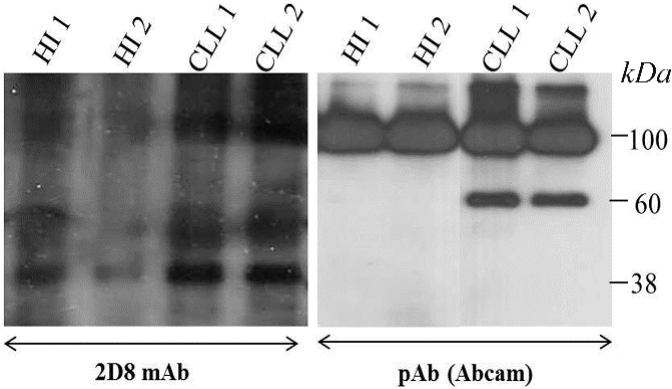
Western blot shows the expression of sortilin in lysate of CLL and healthy PBMCs detected by either 2D8 mAb (left) or antisortilin pAb (Abcam, right). HI: healthy individual.

To the best of our knowledge, this is the first time reporting cell surface expression of sortilin in malignant B cells of CLL patients. We used three different protein readout systems to verify our findings. Flow cytometry results shows an ectopic expression of sortilin by localizing the protein on the surface of malignant B cells which differs from its subcellular localization either intracellular or secreted forms in normal B cells 18. The Western blot data using 2D8 mAb (Figure 6) clearly shows expression of a 95 *kDa* band in CLL lysates but not in normal healthy B cells (left panel) confirming our immunocytochemistry data in normal B cells (Figure 3). Additionally, our antibody detects a strong band of 40 *kDa* in CLL cells with a faint band in normal B cells.

Furthermore, using a commercially available anti-sortilin antibody in Western blot experiments a 60 *kDa* band of sortilin variant (in addition to the 95 *kDa* band) was observed in CLL cells but not in normal healthy individuals (Figure 6, right panel). This may suggest that the structure of sortilin protein expressed in malignant B cells differs from its normal counterpart. Such difference has been reported for ectopic expression of a 38 *kDa* proline/arginine rich end leucine-rich repeat protein (PRELP) in leukemic B cells from CLL patients 19. It seems that variation in the structure of ectopically expressed proteins may affect the subcellular localization, representing a hallmark of cancer cells. This notion should be considered in discovery of new markers targeting cancer.

Here we report overexpression of sortilin on the surface of leukemic B cells in comparison with healthy PBMCs. Therefore, sortilin might be categorized as a diagnostic biomarker in CLL patients. As there was no significant difference in sortilin expression between two groups of progressive and non-progressive cases, it may not be classified as a prognostic indicator of CLL. This might imply that sortilin expression is a genuine phenomenon of malignant B cells initiated since the formation of leukemic cells.

Expression of sortilin was studied in different stages (0, I and II) of CLL; however, no statistically difference was found. Perhaps more comprehensive studies could assist in defining the sortilin as an adequate staging marker in CLL patients. We found a significant relationship between expression of sortilin and CD23 as an immunophenotypic marker of CLL. Since CD23 contributes to accumulation of long-lived B CLL cells 20, it may conclude that sortilin can play a role in B cell survival akin to CD23. CD23 is a cell surface protein considered to be important in differentiation of CLL from Mantle Cell Lymphoma (MCL) and Marginal Zone Lymphoma (MZL) 21. Therefore, it is wise to investigate the lack/presence of sortilin in patients with MCL and MZL; which may add another tumor marker to the current diagnostic panel for differentiating CLL from other Non-Hodgkin’s lymphomas.

Although it is not obvious why CLL cells harbor cell surface sortilin, several studies suggest that sortilin may also undertake vital tasks in the pathophysiology of other cancers. Recent finding suggests a role of sortilin in angiogenesis through releasing of exosomes to the extracellular media 22. A complex composed of sortilin and two tyrosine kinase receptors containing TrkB and EGFR in exosome structure mediated communication and signaling events between the lung cancer cells and its target, the endothelial cells 22. Theimmunohistochemistry experiments performed in a cohort of breast cancers patients showed an increase in the expression of sortilin which was associated with the breast cancer aggressiveness and invasion 5. In other study, it was shown that activation of matrix metalloproteinases in HT29 colorectal cancer cell line led to cleavage of the luminal part of sortilin resulting in release of its soluble form (sSortilin) 23. Subsequently, activation of Focal Adhesion Kinase pathway by sSortilin regulates numerous downstream intracellular pathways mediating either cell migration or metastasis 23. Sortilin as a neurotrophin transporter is implicated in cell proliferation and anti-apoptotic effect of Brain-Derived Neurotrophic Factor (BDNF) in colorectal cancer cell 3. The phenomenon is accelerated by high affinity binding of sortilin to pro-BDNF as an apoptosis inducer in this type of malignancy. Knocking down of sortilin using siRNA treatment showed that sortilin plays a survival role in Caov-4 ovarian carcinoma cell line 13. In the same way, sortilin silencing resulted inhibition of cell survival and migration through decreased activation of extracellular signal-regulated kinases (ERK) in CAL-62 thyroid cancer cell line 24.

To investigate the potency of sortilin as a therapeutic biomarker in CLL, we explored the apoptosis induction by targeting sortilin using 2D8 mAb. As shown in figure 5A, treatment with 2D8 mAb considerably led to apoptosis in CLL cells but not in healthy PBMCs (p≤0.01). It is not exactly clear how 2D8 mAb induces cell destruction; however, it is obvious that this antibody directly induces apoptosis in CLL PBMCs. Mechanisms like antigen crosslinking, blockade of ligand-receptor growth or survival pathways, or activation of death receptors potentially may elicit the induction of direct apoptosis in targeted cells 25. Previously, it was shown that sortilin forms a functional complex by heterodimerizion with Neurotensin Receptor 1 (NT-R1), mediating internalization of neurotensin growth signaling pathways 26,27. Therefore, it is also possible that 2D8 mAb may induce direct apoptosis in CLL cells by blockade of this type of heterodimerzation. Therefore, it is necessary to elucidate the expression of NTR1, its heterodimarization with NTR3, and also endogenous expression of neurotensin in CLL cells to firmly state this hypothesis. In overall, beyond the mechanism of action, mAbs can induce targeted cell killing alone or can enhance target cell susceptibility to chemo- or radio-therapeutics by affecting the modulation of anti-apoptotic pathways 25.

Here we suggest a combination of chemo- and immunotherapy regimens containing specific mAbs targeting sortilin which might increase the efficacy of responses in CLL. Akin to the improved response achieved from combination of anti-CD20 antibody (of atumumab) plus fludarabine and cyclophosphamide in patients with relapsed CLL 28. Since CLL is a complex malignancy with a very heterogeneous pattern, and many signaling pathways may act in parallel in cancer cells; a combination of multi-targeting antibody-based therapy is necessary 29.

Based on a report on expression profile of sortilin in normal resting B cells and few malignant B cell lines, no surface expression of sortilin was detected either in normal or malignant B cells 18. Although sortilin is expressed in normal B cells but its localization differs in normal and malignant conditions. Our results clearly show that our anti-sortilin antibody detects sortilin on the surface of malignant B cells. Such discrepancy in findings might be due to the nature of immunogen used for antibody generation, as our antibody generated against the very N-terminal part of sortilin encompassing signal peptide 12.

## Conclusion

In conclusion, our results from apoptosis induction in CLL cells by targeting sortilin might be attributed to the significant role of sortilin in CLL survival. Therefore, we believe that this is an important finding, which may potentially contribute to diagnosis, monitoring, and therapy of patients with CLL.
